# Outcomes of implementing a coaching-based WHO Safe Childbirth Checklist program in India

**DOI:** 10.1056/NEJMoa1701075

**Published:** 2017-12-14

**Authors:** Katherine E. A. Semrau, Lisa R. Hirschhorn, Megan Marx Delaney, Vinay Pratap Singh, Rajiv Saurastri, Narender Sharma, Danielle E. Tuller, Rebecca Firestone, Stuart Lipsitz, Neelam Dhingra-Kumar, Bhalachandra Kodkany, Vishwajeet Kumar, Atul A. Gawande

**Affiliations:** 1 Ariadne Labs | Brigham & Women’s Hospital, Harvard T.H. Chan School of Public Health, Boston, MA, USA; 2 Division of Global Health Equity, Brigham & Women’s Hospital, Boston, MA, USA; 3 Department of Medicine, Harvard Medical School, Boston, MA, USA; 4 Feinberg School of Medicine, Northwestern University, Chicago, IL, USA,; 5 Population Services International, Lucknow, India; 6 Population Services International, Washington, DC, USA; 7 World Health Organization, Geneva, Switzerland; 8 Jawaharlal Nehru Medical College, Belgaum, India; 9 Community Empowerment Lab, Lucknow, India; 10 Department of Surgery, Brigham & Women’s Hospital, Boston, MA, USA; 11 Department of Health Policy & Management, Harvard T.H. Chan School of Public Health, Boston, MA, USA; 12 Population Services International, New Delhi, India; 13 Center for Health Decision Sciences, Harvard T.H. Chan School of Public Health, Boston, MA, USA

**Keywords:** Essential Birth Practices, Early Neonatal Mortality, Perinatal Mortality, Behavior Change, Birth Attendants, Randomized Controlled Trial, Coaching, WHO Safe Childbirth Checklist, Maternal Mortality, Maternal Morbidity, India, Uttar Pradesh, Public Health Facility

## Abstract

**Background:**

Facility-based childbirth in low-resource settings has increased dramatically over the last two decades, yet quality of care gaps persist and mortality rates remain high. The World Health Organization (WHO) Safe Childbirth Checklist, a quality improvement tool, promotes systematic adherence to practices known to save lives and prevent harm during childbirth.

**Methods:**

We conducted a matched-pair, cluster-randomized controlled trial in 60 pairs o facilities across 24 districts of Uttar Pradesh, India to test the effectiveness of the BetterBirth program, an 8-month coaching-based implementation of the Checklist, on a composite outcome of 7-day maternal/perinatal mortality and maternal morbidity. Outcomes—assessed 8-42 days post-partum—were compared between study arms adjusting for clustering and matching. We also compared birth attendants’ mean adherence to 18 essential birth practices in 15 matched pairs of facilities at 2 and 12 months after intervention initiation.

**Results:**

Of 161,107 eligible women, we enrolled 157,689 (98%) and determined 7-day outcomes for 157,145 (99.7%) mother-newborn dyads. Of 4888 observed births, birth attendants’ adherence to practices was significantly higher in the intervention (I) than control (C) arm (I: 73% vs. C: 42% at 2 months, p≤0.01; I: 62% vs. C: 44% at 12 months, p≤0.01). However, we found no difference in the composite outcome (I: 15.1% C: 15.3%, RR: 0.99, 95% CI: 0.83-1.18, p=0.90).

**Conclusion:**

The coaching-based WHO Safe Childbirth Checklist program produced increased adherence to some essential birth practices, but did not reduce morbidity and mortality. (Clinical Trials #NCT02148952; The Bill & Melinda Gates Foundation)

## Introduction

Globally, maternal mortality ratios range from 3 to 1360 per 100,000 births; neonatal mortality rates from 0.95 to 40.6 per 1000; and stillbirths from 1.2 to 56.3 per 1000, with low- and middle- income countries experiencing rates an order of magnitude higher than high-income regions.^1^,^2^ While we have made progress in recent decades, there is substantial room for improvement.^1^,^3-5^ Despite a dramatic shift from home to facility-based births, birth attendants often do not perform practices known to reduce mortality and mortality rates have not decreased as expected.^6^

Research has shown that programs solely seeking to strengthen birth attendants’ training or to improve supply availability are insufficient to meaningfully improve patient care or outcomes.^7^ Conversely, interventions incorporating job aids and on-site coaching have proven effective in improving individual clinical practices, such as newborn resuscitation or active management of the third stage of labor, as well as outcomes.^8-12^ To bridge the gap between evidence and practice, the World Health Organization (WHO) created the Safe Childbirth Checklist, a practical tool to assist birth attendants in planning for and performing a more complete bundle of 28 essential birth practices.^13^,^14^ These key practices address the most common causes of avoidable mortality for women and newborns.^15^

**Figure 1:**
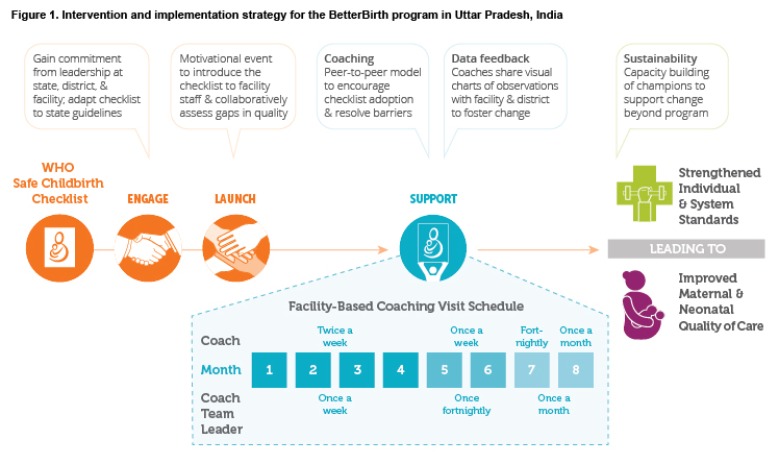
Intervention and implementation strategy for the BetterBirth program in Uttar Pradesh, India

Studies have previously shown that, when well implemented at a small scale, the WHO Safe Childbirth Checklist improves facility-based birth attendants’ adherence to evidence-based care.^16-18^ We tested the BetterBirth program, a coaching-based implementation of the Checklist, in a large-scale, matched-pair, cluster-randomized controlled trial in Uttar Pradesh, India.^19^ We intended our intervention to support providers at multiple levels of the health system in using the Checklist appropriately, to identify gaps in facilities’ quality of care, and to activate resources (e.g. skills training and supply provision) within the existing healthcare system to address these gaps (Figure 1). We hypothesized that the intervention, implemented at the facility (cluster level), would improve quality of care in facility-based childbirth by increasing birth attendants’ adherence to Checklist practices. Further, we hypothesized that this increase in adherence to practices would lead to a decrease in our composite outcome, consisting of maternal mortality [day 0-7], maternal severe morbidity [day 0-7], early neonatal mortality [day 0-7], and stillbirth.

## Methods

### Trial Design

We conducted a matched-pair, cluster-randomized controlled trial in government health facilities, which received either the BetterBirth program, a coaching-based implementation of the WHO Safe Childbirth Checklist (60 facilities; Figure 1) or the existing standard of care (60 facilities). We described the methodology of the BetterBirth Trial^19^, the BetterBirth Program/intervention^20^,^21^, and our Data Quality Assurance system^22^ elsewhere.

### Study Setting and Participants

The most populous state in India (204 million, 77% rural),^23^ Uttar Pradesh is a high-priority region for national and international public-health organizations due to its persistently high neonatal mortality rate (25 per 1000) and maternal mortality ratio (258 per 100,000).^24^,^25^

The Government of Uttar Pradesh permitted the trial to proceed in 38 districts, where we identified 320 eligible facilities. We considered a facility eligible if it was designated as a Primary, Community, or First Referral Unit Health Center; had >=1000 deliveries annually; had >=3 birth attendants trained as auxiliary nurse midwives (or higher); had no other concurrent quality improvement or research programs; and had district and facility leadership willing to participate. The final study sample included 120 facilities across 24 districts (Supplement 1).

We matched facilities (i.e. clusters) on the following criteria prior to randomization: geographic zone, functional classification, distance to a district hospital, annual birth volume, and number of birth attendants (Supplement 1). We randomized facilities to study arm within the matched pair (Figure 2). After matching and randomization, we collected baseline practice-adherence data in 10 sites to confirm successful matching (Figure 2).

Women registered for labor and delivery—excluding women who delivered outside the facility, women who were referred in from another facility, or women who were managed for abortion— were eligible for the study. At each intervention facility and its matched control site, we began enrolling patients 2 months after intervention initiation. Enrollment continued until the site’s target sample size was reached or for 24 weeks after intervention completion, whichever occurred first with a 12-week minimum follow-up.

### Intervention

We implemented the BetterBirth program following the Engage-Launch-Support model (Figure 1) piloted at non-study sites in Karnataka and Uttar Pradesh, India.^18^,^21^,^26^ Coaches (nurses) and Coach Team Leaders (physicians or public-health professionals), unaffiliated with facilities and comprehensively trained to apply a standard behavior-change framework, conducted site visits over the 8-month Support phase.^20^,^21^ We expected Coaches to conduct 43 day-long visits to each facility, beginning twice-weekly and tapering to monthly visits. Coach Team Leaders accompanied Coaches on alternating visits (23 total visits) (Figure 1). Each facility chose at least one staff member to serve as a Childbirth Quality Coordinator, a local champion for the use of the Checklist and continued coaching. 

Coaches motivated birth attendants to use the Checklist and to identify, understand, and resolve barriers to providing quality care.^20^,^21^ Coach Team Leaders supported facility leadership in fostering team communication and addressing gaps in care at facility and district levels by accessing resources through the established healthcare system. Cloud-based data collection enabled rapid feedback on a facility’s progress. We provided no clinical-skills training, financial support, or clinical supplies (except paper copies of the Checklist).

### Data Collection and Outcomes

We measured a composite outcome of events occurring within the first 7-days postpartum, incorporating stillbirth; early neonatal death; maternal death; or self-reported maternal severe morbidity, including seizures, loss of consciousness for >1 hour, fever with foul-smelling vaginal discharge, hemorrhage, or stroke. We selected morbidity measures based on the WHO maternal near-miss approach using questions previously validated for self-report.^27-30^ We calculated an additional composite outcome consisting of mortality events only.

Secondary maternal outcomes by 7-days postpartum included maternal mortality, maternal morbidity, inter-facility transfer (referral), cesarean section, hysterectomy, blood transfusion, and return to the facility for a health problem. Secondary newborn outcomes included stillbirth, early neonatal death, and inter-facility transfer. We assessed all outcomes from facility register information and by contacting women or close family members by telephone between 8 and 22 days post-partum. If we received no response by 22 days post-partum, a fieldworker conducted a home visit.

Additionally, we selected a convenience sample of 15 matched pairs of facilities in which trained nurse-data-collectors directly observed birth attendants providing care over a 12-hour (daytime) shift at 2 months and 12 months after intervention initiation (4 months after coaching cessation). These independent observers measured practice adherence, including supply availability (Table 2). Intervention staff and independent observers were not present at the same facility simultaneously.

Due to the nature of the intervention, we were unable to blind any facility staff, most trial staff, or any investigators to the identity of intervention and control facilities. Call center staff, who collected the majority of outcome data, were blinded to facility assignment.

### Sample Size

*A priori*, we hypothesized a 15% reduction in the primary composite outcome in the intervention arm. We estimated the intracluster (within facility) correlation (ICC) to be 0.01 and the matching effect to reduce the standard error by 45%, basing parameters on previous studies.^31^ We aimed to enroll 171,964 women (85,982 per arm) to detect a decrease from 60 events per 1000 births (control arm) to 51 events per 1000 births (intervention arm) with 80% power and alpha=0.05.

Based on limited data available from Uttar Pradesh, the baseline rate of the primary composite outcome may have been as high as 120 events per 1000 births. The baseline rate used in calculations was purposively set lower than the estimated due to limited information as well as inclusion of community-based birth events in the available data, which may have elevated mortality rates.

In assessing practice adherence, we assumed an ICC (within facility) of 0.01 and a design effect of matching of 0.75. With 15 matched pairs, we had more than 80% power at alpha=0.05 to detect an absolute difference of 8.5% in any birth practice between study arms.

### Statistical Methods

Using an intent-to-treat approach, we compared outcomes between study arms using a Rao- Scott chi-square test, accounting for the matched-pair, cluster design.^32^ The main outcome was the dichotomous composite outcome that was present if any of the 3 main outcomes occurred (maternal mortality, stillbirth/early neonatal mortality, or maternal severe morbidity). This variable was then used to estimate a composite relative risk.^33,34^ An additional secondary composite outcome included maternal and perinatal mortality only. In secondary analyses, each of the main outcomes were compared across arms; a Rao-Scott 3-degree-of-freedom test was used to assess the overall causal effect. No adjustment for multiplicity of testing was made. 

For the subset of directly observed births, we calculated adherence frequencies for each measured practice at 2 months and 12 months after intervention initiation. Further, we calculated an aggregate adherence score by summing the total number of 18 non-conditional practices performed in each delivery (Supplement 1). We generated the mean number of practices (presented as a fraction of 18) performed in each study arm and compared the differences at each time point, using a Rao-Scott chi-square test.^32^ For comparison of individual practices, we used a bias-corrected logistic regression approach that can handle zero cells and complete separation of points within strata and clusters.^35^ We conducted all statistical analyses using SAS v9.4 (SAS Institute, Cary, NC).

### Ethical Compliance

Each facility’s leadership provided facility-level consent for participation and permission for trial staff to collect de-identified data on every eligible woman from facility registers. Before patient discharge, we obtained verbal informed consent and contact information from each woman or her surrogate for follow-up. Data collectors reconfirmed verbal consent at start of the follow-up call or visit. In directly observed births, women or their surrogates provided written consent for observation.

At trial initiation, birth attendants and facility staff verbally agreed to participate. Before an independent observer collected data, the birth attendant verbally reconfirmed agreement. Electronic data were de-identified and stored in a HIPAA-compliant database to ensure participant privacy.

The study protocol was approved by the following ethical review boards: Community Empowerment Lab (Ref no: 2014006), Jawaharlal Nehru Medical College (Ref no: MDC/IECHSR/2015-16/A-53), Harvard T.H. Chan School of Public Health (Protocol 21975-102), Population Services International (Protocol ID: 47.2012), World Health Organization (Protocol ID: RPC 501), and Indian Council of Medical Research. The protocol was reviewed and reapproved on an annual basis. A Data Safety Monitoring Board (DSMB) met every 6 months after enrollment initiation, including interim-analysis when 30% of data was collected (Supplement 1).

## Results

### Facility and Patient Characteristics

All 120 matched and randomized facilities initiated the study. During data collection, 2 facilities closed for renovations, halting enrollment prematurely for those facilities and their matched pairs. Of the 163,939 women registered for labor and delivery (83,166 intervention; 80,773 control), 98.3% (161,107) were eligible for study inclusion and 97.9% (157,689) consented (Figure 2). We collected 7-day outcomes for all but 544 (0.3%) of the consented women. We found no significant differences between intervention and control arms in facility, maternal, or newborn characteristics (Table 1).

The BetterBirth Program was successfully implemented in all 60 intervention facilities, achieving high fidelity to the expected number of coaching visits (average 42.1 visits of 43 expected), interactions with facility leadership (average 14.8 interactions of 11 expected), and facility data- sharing meetings (average 8.6 meetings of 11 expected).

### Adherence to Birth Practices

After 2 months of twice-weekly coaching, birth attendants in intervention facilities (1259 observations) performed 73% of measured practices compared to 42% in control facility attendants (1304 observations) (p≤0.01) (Table 2). Birth attendants performed specific practices, such as blood pressure and temperature assessment, proper hand hygiene, and early newborn care, at significantly higher rates in the intervention arm. Supply availability was similar between study arms. In intervention sites, the Checklist was used at admission in 56.8% of observed births and in 74.3% of births within the first hour post-partum.

Although adherence in intervention facilities remained significantly higher than control facilities, adherence in the intervention arm decreased to 62% of practices per childbirth at 12-months, four months after coaching ceased. For example, administration of oxytocin soon after delivery decreased by nearly one third (from 79.5% to 53.9%) between 2 and 12 months. Similarly, Checklist use declined in intervention sites at 12 months (17.4% at admission; 35.1% within 1 hour post-partum). In control sites, overall birth attendant adherence remained low at both 2 and 12 months (42% and 44%, respectively).

### Mortality and Morbidity

We found no significant difference between intervention and control facilities in our primary outcome (Intervention 15.1%, Control 15.3%, RR: 0.99, 95% CI: 0.83-1.18, p=0.90) or in any secondary outcomes (Table 3). We found no difference in the rates of follow-up care required for women or newborns, hysterectomy, blood transfusion, or inter-facility transfer (referral) for women or newborns. In stratified analyses, we observed no differences between arms based on phase of the intervention (intensive coaching, tapered coaching, and four months post- intervention), time of delivery, or in-facility mortality rate (data not shown).

## Discussion

Implementation of the WHO Safe Childbirth Checklist and similarly constructed tools have suggested impact on quality of care, but have lacked rigorous data evaluating both adherence to essential birth practices and morbidity and mortality.^11^,^12^,^16-18^ In this large matched-pair, cluster-randomized controlled trial in Uttar Pradesh, India, we found that the BetterBirth Program—a coaching-based implementation of the WHO Safe Childbirth Checklist— demonstrated no impact on our primary composite maternal/perinatal health outcome (nor on any secondary health outcomes), despite significant improvement in birth attendant adherence to essential practices in intervention facilities.

The majority of maternal and neonatal mortality happens around the time of birth and within the first 7 days postpartum;^3^,^36^ thus, interventions to improve early outcomes are desperately needed. The BetterBirth Program’s theory of change—that improving the quality of childbirth- related care provided in facilities would translate into improved patient outcomes—reflects basic assumptions underlying current childbirth work in global health. We found that the largely rural population living in this resource-limited setting had a perinatal mortality rate (47 per 1000) and a maternal morbidity rate (12%) much higher than anticipated;^25^ and event rates varied widely across facilities, with up to 10-fold differences observed (Supplement 1). Quality of care in control sites, as measured through birth attendant adherence to practices, was far lower than previously recognized.^16^,^25^,^37-41^ Overall, birth attendants in non-intervention facilities performed just 40% of measured essential practices in a typical birth, such as appropriate hand hygiene (used in >1% of deliveries) or administration of oxytocin within the first minute postpartum to reduce hemorrhage (used in >25%).

We appeared to achieve the first step in the theory of change, demonstrating that a coaching- based implementation of the Checklist could produce broad-based improvement in the quality of care of facility-based childbirth. In intervention facilities, birth attendants substantially increased their performance of measured practices. At two months, intervention sites saw significant improvement in almost all of the practices that were rarely performed in control sites. Staff at intervention facilities had a corresponding increase in Checklist use during the coaching intervention. However, overall levels of adherence and Checklist use diminished after coaching ceased, and some practices never improved compared to controls. It is possible that Checklist use was not sustained due to lack of Checklist stock, staff belief that they knew the items on the Checklist, lack of enthusiasm, or other reasons. While we achieved relative success in improving the quality of care delivered in int

A potential conclusion from our findings is that increasing adherence to these practices is not a worthwhile goal, as these practices did not lead to improved outcomes. We strongly believe this conclusion to be false. Each of the practices incorporated in the Checklist (and therefore in the BetterBirth Program) has its own evidence base, including effectiveness on improving maternal and/or neonatal outcomes.^13^,^15^ At least one practice (proper hand hygiene) has evidence of saving mothers and newborns stretching as far back as the 1840s.^42^

Several other factors may have affected the outcome. The measured levels of improvement in adherence to essential birth practices may not have reached sufficient levels to affect outcomes. For instance, hand hygiene reached only 35% adherence; although skin-to-skin initiation was 79%, maintenance at one hour was just 19%; and magnesium sulfate administration did not increase despite improved maternal blood pressure measurement. The measured levels of adherence may have been misleading, if staff practiced markedly differently when unobserved.^43^ However, we compared treatment effects between sites with and without observation, finding no differences. Persistent gaps in technical skills, complication management, quality as well as quantity of supplies and medicines, access to supportive management and systems level accountability—which were mostly unmeasured—could also have had a significant impact on the ability to improve health outcomes. Factors not targeted by the BetterBirth Program may have also limited the impact, including women’s underlying health and nutrition status, the quality of pre- and post-natal care, and the quality of referral care for those with more complex needs.

In sum, we found that a coaching-based implementation of the WHO Safe Childbirth Checklist appeared to drive substantial improvements in the quality of facility-based childbirth care, but did not achieve impact on adverse maternal and perinatal health outcomes. High-quality research on large-scale childbirth improvement programs is feasible; such studies must continue to measure both processes and outcomes of care. Further investigation is required to understand and modify the complex interaction between quality of care, morbidity, and mortality.

## Supplementary Appendix

Supplementary Material

## Figures and Tables

**Figure:**
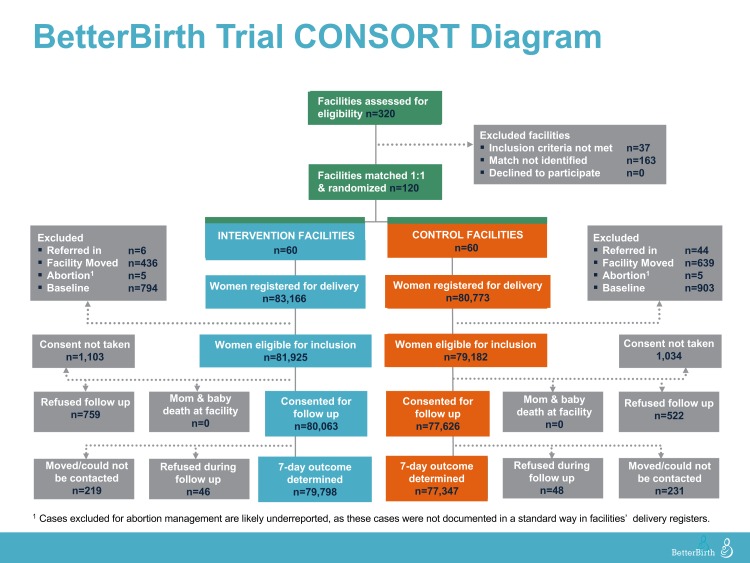
BetterBirth Trial CONSORT Diagram
