# Supplementary material for: Outcomes of implementing a coaching-based WHO Safe Childbirth Checklist program in India
Source: N Engl J Med. Author manuscript; Available in PMC 2017 Dec 14. (PMC5672590; doi:10.1056/NEJMoa1701075)
Supplement: Supplementary Material [file NEJMoa1701075_Semrau_SuppApp.pdf]

Supplementary Appendix for “Outcomes of implementing a coaching-based WHO Safe Childbirth Checklist program in India”

Table of Contents:

Site selection and matching 1
Sample size calculation........ 2
Determination of 18 key practices for adherence assessment 3
Data Safety and Monitoring Board 3
Table 1: Facility and participant characteristics of the BetterBirth trial in Uttar Pradesh, India 4
Figure 1: Variation of maternal and perinatal health outcomes at the facility level in the BetterBirth trial in Uttar Pradesh, India (n=120 sites)........ 6

Site selection and matching

Site selection from the 320 potential sites across 38 districts was narrowed to 120 sites through the matching process. Thirty-seven facilities did not meet the inclusion criteria for study participation; those criteria were facilities with: (1) a designation as a Primary, Community, or First Referral Unit Health Center; (2) ≥1000 deliveries annually; (3) ≥3 birth attendants trained as auxiliary nurse midwives (or higher); (4) no other concurrent quality improvement or research programs; and (5) district and facility leadership willing to participate.

Each facility, meeting the above criteria, was matched to another facility using the following parameters: (1) geographic zone/location; (2) same classification/type (note community health centers and first referral units were considered equivalent); (3) average delivery load +/- 500 deliveries; (4) staffing total +/- 2 attendants; (5) closest single facility on distance to district hospital. All facilities were run through the matching algorithm and matches were set aside. The algorithm was then again run on non-matched facilities. We did not relax our matching criteria to find additional matches. Rather, we used all available matched and selected the first 60 pairs.

We compared sites that were matched and enrolled in the trial compared to the sites that were not matched. There was a significantly higher delivery load in non-matched sites compared to matched sites (p<0.01), but other matching characteristics were similar between matched and no-matched sites.

	Matched Sites	Non-matched Sites	Ineligible Sites
Facility Type			
CHC/FRU	10.2%	15.0%	4.8%
CHC	58.2%	58.9%	35.7%
PHC	31.6%	26.1%	45.2%
District Hospital	0%	0%	4.8%
Sub-Center	0%	0%	9.5%
Annual delivery load (mean ±SD)*	1710.6 (54)	2189 (65)	788 (98)
Number of staff birth attendants (mean±SD)	4.4 (0.2)	4.0 (0.1)	2.7 (0.3)
Distance to district hospital (mean±SD)	31.52 (1.5)	31.85 (1.3)	36.5 (2.8)

*Annual delivery loads are significantly higher in non-matched sites c

Sample Size Calculation

In the design phase of the trial, sample size calculations were based on the assumption that the event rate of the composite outcome would be 60 events per 1000 births. The primary outcome in this trial was the rate of the composite measure of maternal death within 7 days, severe maternal complications within 7 days, fresh or macerated stillbirth, and neonatal death within 7 days. The challenge we faced in determining a baseline rate for the composite outcome is outlined in the BetterBirth Protocol. In 2011, data available from Uttar Pradesh suggested that the baseline rate of the primary outcome would be no more than 50-60/1,000 live births. Preliminary data from the study collected during the pilot phases suggest that the baseline rate may actually be greater than 100/1,000. The baseline rate used in the sample size calculations was set at 60 events/1,000. This was purposively set lower than the pilot data to (1) maintain conservatism since the preliminary data is based on a small sample and we do not want to risk under powering the study; (2) acknowledge that preliminary data may have included community-based events, which may have elevated mortality rates; and (3) facilitate the possibility of demonstrating significant changes in sub-analyses (i.e., perinatal death rate).

For sample size calculations, we estimated intra-cluster correlation (ICC) to 0.01. Upon completion of the study, we calculated the ICC for the composite and individual outcomes. The ICC found in the study are reported below:

Outcome	ICC (95% CI)
Composite	0.018820 (0.003451333, 0.096033)
Perinatal mortality	0.005378 (0.004087,0.007398)
Maternal mortality	0.000245 (0.000109,0.000958)
Maternal morbidity	0.01963 (0.01531,0.02606)

Determination of 18 key practices for adherence assessment

Independent nurse data collectors observed and documented care provided by birth attendants in 30 facilities (15 matched pairs) at 2 and 12 months after the intervention started. Measured behaviors were based on items from the Checklist, but operationalized so that data collectors could observe adherence when standing nearby, without interfering or interrupting care. During their observations, data collectors assessed adherence to practices as well as supply availability from the time of the woman's admission to the facility through the first hour after delivery. All practices measured are reported in Table 2 in the manuscript.

Of the practices measured, 18 are key behaviors that should be conducted or essential supplies that should be available for all women and newborns during childbirth. We created a summary score of adherence to those key 18 practices. We calculated the score as a percent adherence out of the 18 practices; we calculated the average (mean) of that adherence level and reported it as a proportion. The behaviors and supplies were not conditional on appropriate administration. For example, provision of magnesium sulfate is not included in this summary score of 18 as those medicines should only be provided in specific cases.

Data Safety and Monitoring Board

A Data Safety Monitoring Board (DSMB), consisting of Dr. Cyrus Mehta (Chair), Dr. Shally Awasthi, and Dr. Nozer Sheriar, met every 6 months after enrollment initiation and

conducted an interim-analysis review when 30% of data was collected. For the interim analysis, the Haybittle-Peto[1] stopping rule was used with a p-value <0.001 for our composite measure comparing study arms. In addition, the DSMB reviewed women's complication rates and birth attendants' adherence to the Checklist. With the Haybittle-Peto approach, it is appropriate to use the conventional significance level of p-value <0.05 in the final analysis.

Table 1: Facility and participant characteristics of the BetterBirth trial in Uttar Pradesh, India

Here we present an expanded demographic characteristic table for readers to further understand the context in which the trial was operating.

	Intervention		Control	
**Facility Matching Criteria**				
Number of facilities (n)	60		60	
Annual delivery load (mean, 95% CI)	1598.9	1485.9-1711.8	1683.1	1552.0-1814.2
Functional Classification of facility (n, %)				
Primary Health Center	23	38.3%	23	38.3%
Community Health Center	27	45.0%	29	48.3%
First Referral Unit	10	16.7%	8	13.3%
Distance to district hospital in km (mean, 95% CI)	29.5	25.9-33.1	30.3	27.2-33.4
Skilled birth attendants per facility (mean, 95% CI)	4.4	4.1-4.7	4.4	4.1-4.7
**Community Level Characteristics**				
Catchment population per site according to head of each facility in 2016 (mean, 95%CI)	223185	206074-240297	212332	196116-228547
Household income by district in Indian Rupees (mean, 95%CI)[1]	27624	24957-30291	28365	25669-31060
Household income by district in US Dollars (mean, 95%CI)[2]	592.7	535.5-649.9	608.6	550.8-666.4
**Maternal Characteristics**				
Number of women	81925		79182	
Cluster size (mean, 95% CI)	1365.4	1256.4–1474.4	1319.7	1228.9-1410.5
Range		515 - 2697		646 - 2198
Age in years (mean, 95% CI)	25.6	25.5-25.8	25.7	25.5-25.9
Missing age (n, %)	772	0.9%	559	0.7%
Caste (n, %)				
Schedule caste/tribe	23859	29.1%	25175	31.8%
Other backward caste (OBC)	37989	46.4%	35577	44.9%
General caste	13516	16.5%	14122	17.8%
Missing	6561	8.0%	4308	5.4%
Gravida (mean, 95% CI)	2.4	2.3-2.4	2.3	2.3-2.4

Missing (n, %)	16739	20.4%	12853	16.2%
Minutes between admission and delivery (mean, 95% CI)	199.8	183.9-215.7	205.5	190.8-220.2
Median (IQR)	105	33-260	110	35-265
Missing (n, %)	8209	10.0%	7763	9.8%
Admission staffing shift (n, %)				
8:01am to 2pm	24492	29.9%	23083	29.2%
2:01pm to 8pm	17341	21.1%	16814	21.2%
8:01pm to 8am	39504	48.2%	38673	48.8%
Missing	588	0.7%	612	0.8%
Cadre of providers attending delivery based on facility registry (n, %)				
Doctor	11115	13.6%	11599	14.7%
Nurse	66687	81.4%	64117	81.0%
ANM	15311	18.7%	14549	18.4%
Others	2633	3.2%	6560	8.3%
Unknown	3082	3.8%	2483	3.1%
Number of offspring				
Singleton	80402	98.1%	77582	98.0%
Sets of twins	515	0.6%	533	0.7%
Sets of triplets	5	0.01%	5	0.01%
Unknown (referred before delivery)	1003	1.2%	1062	1.3%
**Newborn Characteristics**				
Count of newborns	81447		78663	
Sex of newborn (n, %)				
Male	40558	49.8%	39063	49.7%
Female	36976	45.4%	36266	46.1%
Unknown (referred before delivery)	3913	4.8%	3334	4.2%
Low birth weight defined as 2500 grams or less (n, %)	22316	27.4%	22728	28.9%
Missing (n, %)	5883	7.2%	5510	7.0%
Pre-term birth defined as fewer than 37 weeks gestation (n, %)	15941	19.6%	17703	22.5%
Missing (n, %)	22213	27.3%	19108	24.3%

*No significant differences were observed between study arms using a p-value <0.05 as the cut-off for any variable in this table.

[1] Statistical diary Uttar Pradesh 2014. Economics and Statistics Division, State Planning Institute, Planning Department, Uttar Pradesh. Conversion based on average 2011 rates, available at http://www.x-rates.com/average/?from=USD&to=INR&amount=1&year=2011

[2] Statistical diary Uttar Pradesh 2014. Economics and Statistics Division, State Planning Institute, Planning Department, Uttar Pradesh. Conversion based on average

2011 rates, available at http://www.x-rates.com/average/?from=USD&to=INR&amount=1&year=2011

Figure 1: Variation of maternal and perinatal health outcomes at the facility level in the BetterBirth trial in Uttar Pradesh, India (n=120 sites)

Figure 1 denotes the variation of facility level mortality and morbidity rates. Each dot on the graph represents the facility level outcome with 60 intervention sites and 60 control sites. Variation of health outcomes was extremely high. Perinatal mortality rates were as low as 14 deaths per 1000 births and as high as 104 deaths per 1000 births. Similarly, 7-day maternal mortality ranged from 0 deaths per 100,000 to 470 deaths per 100,000. It is important to note we only had 149 maternal deaths in the entire cohort. Finally, self-reported maternal morbidity, as identified by presence of any of the following: seizures, loss of consciousness for >1 hour, fever with foul-smelling vaginal discharge, hemorrhage, or stroke, ranged from 4.7% to 33%.

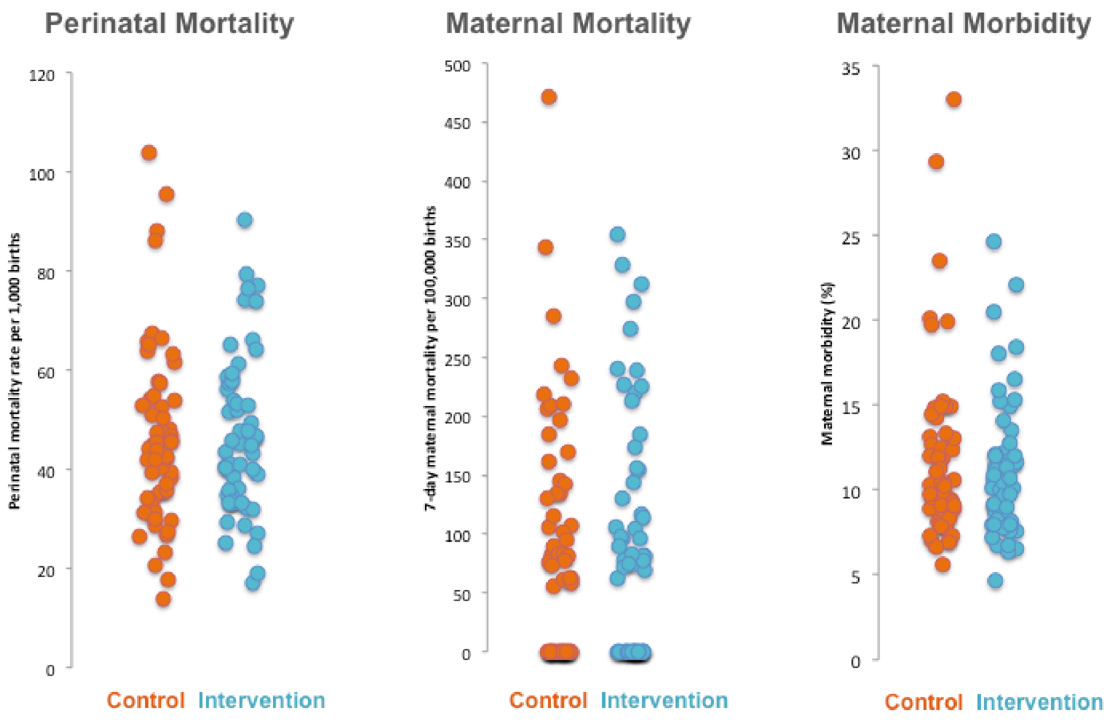

References:

1. Peto R, Pike MC, Armitage P, et al. Design and analysis of randomized clinical trials requiring prolonged observation of each patient. I. Introduction and design. Br J Cancer 1976;34:585-612.
